# Cystatin-C as a Marker for Renal Impairment in Preeclampsia

**DOI:** 10.1155/2017/7406959

**Published:** 2017-07-11

**Authors:** Apeksha Niraula, Madhab Lamsal, Nirmal Baral, Shankar Majhi, Seraj Ahmed Khan, Pritha Basnet, Kashyap Dahal

**Affiliations:** ^1^Department of Biochemistry, B.P. Koirala Institute of Health Sciences, Dharan, Nepal; ^2^Department of Biochemistry, School of Medicine, Xavier University, Oranjestad, Aruba; ^3^Department of Obstetrics and Gynecology, B.P. Koirala Institute of Health Sciences, Dharan, Nepal; ^4^Department of Internal Medicine, B.P. Koirala Institute of Health Sciences, Dharan, Nepal

## Abstract

Preeclampsia is a devastating pregnancy-associated disorder characterized by the onset of hypertension, proteinuria, and edema with limited plausible pathophysiology known. Cystatin-C, a novel marker for the detection of renal impairment, is increased in preeclampsia at an early stage. This study was aimed to evaluate the diagnostic efficiency of Cystatin-C as an early marker of renal function in preeclampsia comparing it to the traditional renal markers. A hospital based comparative cross-sectional study was performed on 104 women (52 diagnosed cases of preeclampsia and 52 healthy pregnant women). Concentrations of Cystatin-C, creatinine, urea, and uric acid were measured in both the study groups. Mean serum Cystatin-C and uric acid levels were elevated in preeclampsia cases compared to controls (1.15 ± 0.37 versus 0.55 ± 0.12; 5.40 ± 1.44 versus 3.97 ± 0.68, resp.). ROC curve depicted that Cystatin-C had the highest diagnostic efficiency (sensitivity, 88.24%; specificity, 98.04%) compared to creatinine and uric acid. Serum Cystatin-C consequently seemed to closely reflect the renal functional changes, which are believed to lead to increased blood pressure levels and urinary excretion of albumin and may thus function as a marker for the stage of the transition between normal adaptive renal changes at term and preeclampsia.

## 1. Introduction

Hypertension is one of the common medical complications of pregnancy and contributes significantly to maternal and perinatal morbidity and mortality [1]. Hypertensive disorders in pregnancy are responsible for 76,000 maternal and 500,000 infant deaths each year worldwide. A World Health Organization (WHO) analysis of maternal deaths reveals that hypertensive disorders are responsible for 16.1% maternal deaths in developed countries and is a major contributor to maternal death in Africa (9.1%) and Asia (9.1%) [2]. Preeclampsia (PE), a multisystem disorder of unknown etiology, is characterized by development of hypertension to the extent of 140/90 mmHg or more with proteinuria after the 20th week in a previously normotensive and nonproteinuric woman with proteinuria [1]. It is defined as maternal systolic blood pressure > 140 mmHg and/or diastolic blood pressure > 90 mmHg measured on two occasions separated by at least 6 hours and proteinuria > 300 mg in a 24-hour period or qualitative, >1+, after 20 weeks of gestation following the guidelines of the American College of Obstetricians and Gynecologists (ACOG), 2002.

The kidneys play an essential role, both in the adaptive physiology of normal pregnancy and in the pathophysiology of PE [3] and some changes in renal function are found to be common to term pregnancy and PE [4]. But, the challenge to every clinician in the present context is to diagnose the renal impairment at an early stage to prevent this leading cause of fetal morbidity and mortality to progress into a severe stage (eclampsia) [5]. Efforts to find an effective predictive test early in pregnancy have not been successful in a low-risk population and there is no gold standard diagnostic test to define PE [6]. The condition is a multisystem disorder, where different aspects of the disease are used in different classifications of hypertensive disorders in pregnancy. This makes it difficult to establish a clear-cut population of women at risk or women with developed PE for investigation and also confuses interpretation of the literature in the field where separate classifications are used [7]. The only consistently found pathological lesion in PE is the renal lesion termed glomerular endotheliosis, which has been regarded as pathognomic for the condition. PE, which is characterized by widespread maternal endothelial dysfunction, inevitably may compromise glomerular dynamics and barrier function [4]. Assessment of renal function is, therefore, important in the evaluation of the pregnant patient with hypertension.

Creatinine is the most widely used biomarker of kidney function but is impervious in the early stages of renal impairment [8]. Serum creatinine levels are elevated in patients with renal malfunction especially with the significant decrease in glomerular filtration. Vasodilation of the renal vessels in pregnancy causes 50–80% increase in plasma flow and change in glomerular filtration rate (GFR), which further complicates the use of serum creatinine as a marker of GFR in pregnancy [9]. Uric acid (UA) is filtered, reabsorbed, and secreted by the kidney. Hypovolemia, an early change in PE, increases UA reabsorption which could increase serum UA concentrations. However, increased UA precedes the reduction in plasma volume [10]. Increased UA production from maternal, fetal, or placental tissues through increased tissues breakdown and/or increased xanthine oxidase activity may also be the cause of increased concentration [11]. Uric acid is also a predictive marker of eclampsia and fetal outcome [12]. Uric acid was popularly used as a marker of GFR when monitoring renal function in PE. Its serum concentration increased with the severity of PE and was assigned as a good predictor in clinical observation, even a pathogenic factor in the pathophysiology of PE. But results of some investigations made UA assessment in the estimation of hypertensive disorder of pregnancy fall into disfavor [13].

All these explanations suggest that these traditional markers of renal function are unable to assess the renal impairment at an early stage and also to detect the reduced GFR in early stages of kidney dysfunction; hence search for new biomarker like Cystatin-C is suggested. In order to overcome this hindrance in estimating renal function in pregnant women, studies have demonstrated that serum Cys-C can reliably reflect the GFR in both healthy and hypertensive pregnant women [13].

Therefore, this study was undertaken to see the diagnostic efficacy of serum Cys-C as a marker of early renal impairment in PE and compare it with other traditional renal markers.

## 2. Aims and Objectives

- To compare serum Cystatin-C, creatinine, and uric acid levels in PE and normal pregnant women.
- To evaluate the diagnostic value of serum Cystatin-C level as an alternative marker of renal function in preeclampsia.

## 3. Methods

The study was conducted at the Department of Obstetrics and Gynecology in collaboration of Department of Biochemistry, BPKIHS, Dharan, Nepal, after being approved by the Institutional Ethical Review Board (IERB), BPKIHS. An informed consent was obtained from all the study participants. Renal function was investigated in two groups of pregnant women: one with preeclampsia (*n* = 51) and the other of healthy pregnant women (*n* = 51).

Blood pressure measurement and urine analysis were performed at the beginning of the pregnancy to exclude preexisting proteinuria or renal disease. Maternal conditions potentially affecting GFR during the study (pregestational hypertension, diabetes, and other concomitant renal diseases), if present, were not included.

The common inclusion criteria for both groups were normal fetal morphology and the absence of concomitant disease and gestation between ≥24 and 36 gestational weeks. Additional inclusion criteria for preeclampsia were a systolic blood pressure level of 140 mmHg or higher or a diastolic blood pressure level of 90 mmHg or higher that occurred after 20 weeks of gestation complicated with proteinuria, defined as the presence of 0.3 g or more of protein in a 24 h urine specimen. Other parameters included in the study were gestational age, parity, and body mass index (BMI). Systolic blood pressure (SBP), diastolic blood pressure (DBP), and mean arterial pressure (MAP) were also noted in both the study groups.

The results from PE group were compared with that of the healthy age and gestational week matched control group.

Serum Cystatin-C levels were measured by Nephelometry method using a Fully Automated Autoanalyser (Accent 2000) with intra- and interassay % CV less than 5% and according to the procedure recommended by reagent manufacturer. Serum creatinine levels were measured by Jaffe's method, in cobas C311 Autoanalyser (Roche Diagnostics) with intra- and interassay % CV less than 2.45% and according to the procedure recommended by the manufacturer. Serum uric acid levels were measured by standardized enzymatic PAP method with uricase and peroxidase, in cobas C311 Autoanalyser with intra- and interassay % CV less than 2.44% and according to the procedure recommended by the manufacturer. Serum urea was estimated by standard urease method in cobas C311 Autoanalyser with intra- and interassay % CV less than 2.44% and according to the procedure recommended by the manufacturer. In patients with PE, total urinary protein levels were measured by modification of the dye binding method used by Fujita et al. [14] and with commercial uric 3V SGO 3100.

## 4. Statistical Analysis

Data were initially checked for normal distribution by Kolmogorov-Smirnov test. For normally distributed data, mean with SD, Pearson's correlation, and Student's *t*-tests were applied. If data was not normally distributed, nonparametric tests were applied. Independent Student's *t*-test was used to compare Cys-C levels, creatinine, urea, and uric acid between the two groups. Pearson's correlation was used to correlate Cys-C levels with POG, BMI, SBP, DBP, MAP, urea, and uric acid in PE and control groups, respectively. Spearman's correlation was used to correlate creatinine and Cys-C levels in PE and control groups, respectively. Multiple linear regression was also performed to explore the association between the predictors, namely, age, POG, BMI, Cys-C, creatinine, urea, and UA and DBP in PE. ROC curve was used to evaluate the diagnostic utility of Cys-C, creatinine, and UA as a marker of renal function in PE.

## 5. Results

Descriptive characteristics of the study participants are illustrated in Table 1. Overall, most of the study participants in both the study groups were multigravida and nullipara. Our study shows that the female developed PE in late gestational period (26.84 ± 5.20). The details of the baseline and clinical parameters have been summarized in Table 1. The mean gestational age of pregnant women with PE was 35.02 ± 4.59. BMI was higher in preeclamptic patients than in control group (28.71 ± 4.30 versus 24.47 ± 2.86). Mean SBP and DBP (mm of Hg) were higher in preeclamptic patients than in control group (146.36 ± 10.67; 112.74 ± 8.21 and 104.31 ± 10.24; 71.76 ± 7.67, resp.). MAP was significantly higher in PE (112.74 ± 8.21) than in control group (82.61 ± 7.86). The details of the baseline and clinical parameters have been summarized in Table 1.

Mean serum Cystatin-C level was higher in PE compared to the control group (1.15 ± 0.37 versus 0.55 ± 0.12) and was statistically significant. Median serum creatinine was higher in PE compared to control group but the distribution between the two groups was statistically insignificant: 0.40 (0.30, 0.50) versus 0.50 (0.35, 0.57). Serum urea was slightly lower in PE compared to control group (15.60 ± 6.10 versus 15.91 ± 5.01) without any statistical significance. Serum uric acid was significantly higher in preeclamptic patients compared to control group; values are 5.40 ± 1.44 versus 3.97 ± 0.68, respectively, as depicted in Table 2. Multiple regression analysis showed that the predictors significantly predict the outcome of DBP and only BMI remained as a single predictor significantly influencing the DBP as illustrated in the table as shown in Table 3. Levels of Cystatin-C have been shown to be unaffected in various age groups. The results of the present study found similar findings; that is, the concentration of serum Cystatin-C was unchanged between various age but the difference was not significant (Table 4).

**Figure 1:**
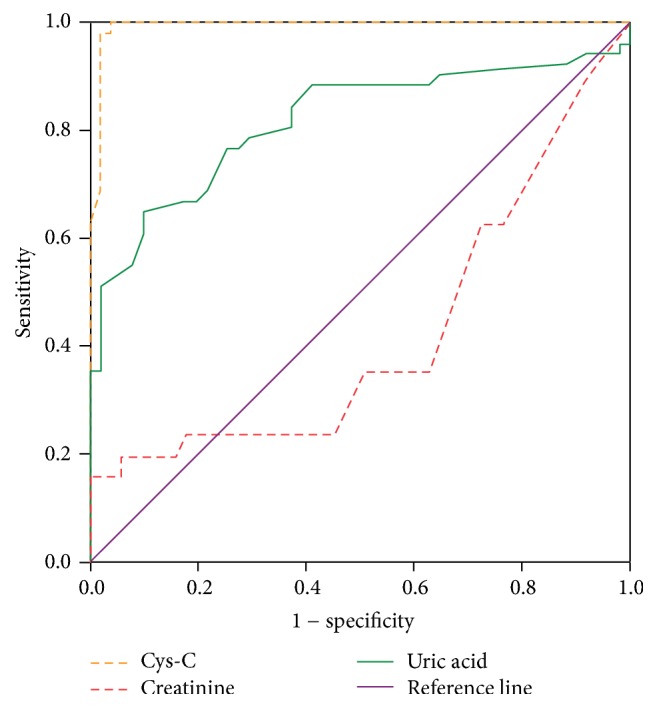
*ROC curve to determine the diagnostic utility of Cystatin-C, creatinine, and uric acid in PE*. Cystatin-C showed the superior diagnostic accuracy compared to the other two traditional renal markers. From the curve, we determined the cut-off value for Cystatin-C with maximum sensitivity and specificity to be 0.9. Similarly, for creatinine, the cut-off was taken as 0.38 and for uric acid 4.25, respectively.

eGFR as calculated by CKD-EPI equation by serum Cystatin- C level (according to KDIGO 2012) was significantly reduced in PE as compared to control group. Mean eGFR in PE was 85.86 ± 28.53 and 130.58 ± 14.22 in control group with the difference being statistically significant. The details have been illustrated in Table 4. Receptor operating characteristics (ROC) curve was used to determine the diagnostic efficacy of three markers, namely, Cys-C, creatinine, and uric acid in PE. Cystatin-C showed the superior diagnostic accuracy compared to the other two traditional renal markers. From the curve, the cut-off value for Cystatin-C was determined with maximum sensitivity and specificity to be 0.9. Similarly, for creatinine, the cut-off was taken as 0.38 and for UA 4.25, respectively. ROC curve has been shown in Figure 1 and sensitivity and specificity of all the three markers have been illustrated (Table 5).

## 6. Discussion

Renal impairment in PE has been implicated to various reasons, the most likely being hemodynamic changes [15] and glomerular endotheliosis [16], as well as podocyte damage [17]. Our study showed that serum Cystatin-C is significantly higher in PE compared to control group. This finding was in accordance with [15, 18, 19]. Preeclamptic patients are at increased risk of renal impairment, though the dysfunction is usually undershadowed during the gestational period. The patients who developed PE are also at increased risk of developing PE in their subsequent pregnancies [20, 21]. The renal impairment if undiagnosed early can progress to renal failure and also lead to other vascular disorders later in life [22–24]. Mean BMI in PE was higher than control group (refer to Table 1). BMI is one among the three risk factors for increased SBP and DBP, the other two being increased maternal age and gestational age. Multiple regression analysis showed that the predictors significantly predict the outcome of DBP and only BMI remained as a single predictor which significantly influenced the DBP as depicted in Table 3. Several investigations have indicated that serum Cystatin-C is a better marker for GFR than serum creatinine, in particular for individuals with small to moderate decreases in GFR [25]; only few investigations regarding Cystatin-C levels in pregnancy have been published [18, 26, 27]. Though, it would have been logical to expect a decreased value of serum Cystatin-C as the renal plasma flow increases during pregnancy leading to about 40% higher GFR, determined as the plasma clearance rate of the low molecular mass substance Iohexol [28]. To this, numerous explanations have been put forward for the fact that serum Cystatin-C does not decrease during pregnancy.

Production of Cystatin-C might be increased during pregnancy due to an increased number of nucleated cells which is supported by a study showing serum Cystatin-C is increased during twin pregnancy [18, 25]. It is more likely that the increase in Cystatin-C levels in pregnancy is due to an altered filtration process than to an increased production rate, as the serum levels of Cystatin-C were not found to correlate to fetal or placental weight in a study conducted by [29] suggesting that Cystatin-C does not cross the placental barrier. Thus, we speculate that there could be a shift towards a more cationic glomerular barrier in pregnant women, resulting in higher serum concentrations of Cystatin-C during pregnancy. Hence, it would be necessary to monitor kidney function during pregnancy by serum Cystatin-C for detection of abnormal kidney function, as serum Cystatin-C seems to reflect an altered filtration process in pregnancy at an early period serum. Cystatin-C is considered to be a superior marker for assessment of renal function and GFR (refer to Table 2) more closely than traditional renal markers, namely, creatinine and uric acid, as Cystatin-C level is not affected by age, gender, race, ethnicity, muscle mass, and diet [30, 31]. The present study analyzed if the level of Cystatin-C varied among the age groups in PE and control group. We did not find any rise in Cystatin-C levels in higher age group, though the difference between the two groups was not statistically significant as shown in Table 4.

As demonstrated by ROC, analysis of the data in the present study is depicted in Figure 1 and Table 5. Serum Cystatin-C showed a highest diagnostic accuracy compared to serum creatinine and serum uric acid [18, 26, 32]. Serum uric acid showed a higher diagnostic accuracy than creatinine and has also been shown to be a useful predictor of fetal outcome in preeclampsia [33–36], though increasing serum levels in PE reflects an enhanced reabsorption in the proximal tubules and not a reduced Cystatin-C [37]. This finding was in accordance with the study done by [18], in which they found that Cystatin-C was higher among all of the PE patients than the control group. Serum creatinine is also of limited use in the assessment of the GFR, which can be reduced by 50% without causing abnormal serum creatinine concentrations [38–40]. Several of the patients with the most severe preeclampsia had normal creatinine levels in our study, whereas all patients with severe preeclampsia had Cystatin-C levels raised above the upper reference limit for normal term pregnancy. Preeclampsia can be diagnosed easily by determining hypertension with proteinuria, but the diagnosis of the true condition associated with the increased risk can still be elusive, as pregnant women can present with hypertension and proteinuria due to other conditions as well, and a preeclamptic state can be present without raised blood pressure or albuminuria [41]. Moreover, blood pressure levels and proteinuria are unstable markers, often varying within a wide range during the course of the disease [42]. The estimation of serum Cystatin-C could be helpful in the diagnosis of PE, reflecting a different feature of the disease as a stable indicator of an altered filtration process and may also prove valuable for the monitoring of GFR in renal disease in pregnancy and in PE [43] (Table 2).

## 7. Conclusion

Preeclampsia is still a leading cause of maternal morbidity and mortality in developed and underdeveloped countries like ours. Renal dysfunction plays a central and initial role in pathophysiology of PE. Hence, assessment of renal function plays a vital role in monitoring and prediction of severity in PE. Thus, an early marker of renal impairment is needed in the diagnosis and thereby preventing progression of PE to eclampsia.

## Figures and Tables

**Table 1:** Baseline and clinical parameters of study participants.

General characteristics	PE (*n* = 51)	Control (*n* = 51)	*p* value
Age (years)	26.84 ± 5.20	25.84 ± 4.54	0.304^a^
POG (weeks)	35.02 ± 4.59	29.35 ± 3.35	0.001^a*∗*^
BMI (Kg/m^2^)	28.71 ± 4.30	24.47 ± 2.86	0.001^a*∗*^
SBP (mmHg)	146.86 ± 10.67	104.31 ± 10.24	0.001^a*∗*^
DBP (mmHg)	95.69 ± 8.77	71.76 ± 7.67	0.001^a*∗*^
MAP	112.74 ± 8.21	8.21 ± 7.86	0.001^a*∗*^

^a^Independent  *t*-test; ^*∗*^*p* value < 0.05 is considered to be significant.

**Table 2:** Biochemical parameters in preeclampsia and control group.

Biochemical parameters	PE (*n* = 51)	Control (*n* = 51)	*p* value
Cystatin-C (mg/L)	1.15 ± 0.37	0.55 ± 0.12	0.001^a*∗*^
Creatinine (mg/dl)	0.4 (0.3, 0.5)	0.5 (0.35, 0.57)	0.178^b^
Urea (mg/dl)	15.60 ± 6.10	15.91 ± 5.01	0.783^a^
Uric acid (mg/dl)	5.40 ± 1.44	3.97 ± 0.68	0.001^a*∗*^

^a^Independent  *t*-test; ^b^Mann–Whitney *U* test (0.4 is the median value and 0.3 and 0.5 represent the 25th and 75th percentile); ^*∗*^*p* value <0.05 is considered to be statistically significant.

**Table 3:** Multiple linear regression analysis of DBP with the ANOVA table for the model.

Variables	Coefficient	SE	*t*-value	*p* value
Intercept	114.55	15.65	—	—
Age (years)	−0.208	0.235	1.488	0.144
POG (weeks)	−0.017	0.289	−0.114	0.910
BMI (kg/m^2^)	−0.420	0.271	−3.166	0.003^*∗*^
Cystatin-C (mg/L)	0.214	0.271	1.434	0.159
Creatinine (mg/dl)	0.119	2.132	0.904	0.371
Urea (mg/dl)	0.121	0.189	0.922	0.362
Uric acid (mg/dl)	0.207	0.851	1.475	0.148

Coefficient: regression coefficient; SE: standard error. ^*∗*^*p* value <0.05 is considered to be statistically significant.

**Table 4:** Comparison of Cystatin-C with various age groups in PE and control group.

Variables	1 (<20 yrs)	2 (20–24 yrs)	3 (25–30 yrs)	4 (>30 yrs)	*p* value
Cystatin-C (mg/L)^a^					
PE	1.10 ± 0.14	1.24 ± 0.39	1.01 ± 0.15	1.23 ± 0.49	0.292
Control group	0.58 ± 0.10	0.55 ± 0.11	0.55 ± 0.11	0.54 ± 0.18	0.978

^a^ANOVA.

**Table 5:** Showing sensitivity and specificity of various renal markers.

Parameters	AUC (95% CI)	Sensitivity	Specificity	+LR	−LR	PPV	NPV
Cystatin-C	0.993	88.24%	98.04%	45.0 (6.4–314.17)	0.12 (0.06–0.25)	97.83%	89.29%
Creatinine	0.423	62.75%	27.45%	0.86 (0.66–1.13)	1.36 (0.77–2.40)	46.38%	42.42%
Uric acid	0.815	79.07%	71.19%	3.78 (2.02–7.05)	0.40 (0.27–0.61)	66.67%	82.35%

There was a significant positive correlation between Cystatin-C level and uric acid (*r* = 0.33; *p* value < 0.05). There was also a significant negative correlation between eGFR and serum Cystatin-C level (*r* = − 0.777, *p* < 0.001). The negative correlation implies that as the eGFR level decreases with the progression of kidney disease, there is a subsequent rise in serum Cystatin-C level.

## References

[1] Dutta D., Konar H. C. (2015). Hypertensive disorders in pregnancy. *DC Dutta’s Textbook of Obstetrics*.

[2] Khan K. S., Wojdyla D., Say L., Gülmezoglu A. M., van Look P. F. (2006). WHO analysis of causes of maternal death: a systematic review. *The Lancet*.

[3] Chesley L. C. (1985). Diagnosis of preeclampsia. *Obstetrics & Gynecology*.

[4] Redman C. W. G., Sacks G. P., Sargent I. L. (1999). Preeclampsia: an excessive maternal inflammatory response to pregnancy. *American Journal of Obstetrics & Gynecology*.

[5] Coppage K. H., Sibai B. M. (2004). Preeclampsia and Eclampsia. *Gynecology and Obstetrics*.

[6] Friedman R., De Azevedo M. J., Gross J. L. (1988). Is endogenous creatinine clearance still a reliable index of glomerular filtration rate in diabetic patients?. *Braz J Med Biol Res*.

[7] Brown M. A., Buddle M. L. (1997). What's in a name? Problems with the classification of hypertension in pregnancy. *Journal of Hypertension*.

[8] Eiland E., Nzerue C., Faulkner M. (2012). Preeclampsia 2012. *Journal of Pregnancy*.

[9] Sumithra K., Vibha C., Vishwanath H. L. (2013). Study of Serum cystatin C in Preeclampsia. *Int J Curr Res*.

[10] Bainbridge S. A., Roberts J. M. (2008). Uric acid as a pathogenic factor in Preeclampsia. *Placenta*.

[11] Gallery E. D. M., Hunyor S. N., Györy A. Z. (1979). Plasma volume contraction: A significant factor in both pregnancy-associated hypertension (preeclampsia) and chronic hypertension in pregnancy. *QJM*.

[12] Lamb E. J., Price C. P. (2013). Kidney Function Tests. *Teitz Textbook of Clinical Chemistry and Molecular Diagnostics*.

[13] Guo H.-X., Wang C.-H., Li Z.-Q. (2012). The application of serum cystatin C in estimating the renal function in women with preeclampsia. *Reproductive Sciences*.

[14] Fujita Y., Mori I., Kitano S. (1983). Color Reaction Between Pyrogallol Red - Molybdenum (VI) Complex and Protein. *Japan Soc Anal Chem*.

[15] Franceschini N., Qiu C., Barrow D. A., Williams M. A. (2008). Cystatin C and preeclampsia: a case control study. *Renal Failure*.

[16] Lafayette R. A., Druzin M., Sibley R. (1998). Nature of glomerular dysfunction in pre-eclampsia. *Kidney International*.

[17] VD Garovic S. J., Wagner Turner S. T. (2007). Urinary podocyte excretion as a marker for preeclampsia. *American Journal of Obstetrics & Gynecology*.

[18] Strevens H., Wide-Swensson D., Grubb A. (2001). Serum cystatin C is a better marker for preeclampsia than serum creatinine or serum urate. *Scandinavian Journal of Clinical and Laboratory Investigation*.

[19] Kristensen K., Wide-Swensson D., Schmidt C. (2007). Cystatin C, *β*-2-microglobulin and *β*-trace protein in pre-eclampsia. *Acta Obstetricia et Gynecologica Scandinavica*.

[20] Dekker G. A., Sibai B. M. (1998). Etiology and pathogenesis of preeclampsia: Current concepts. *American Journal of Obstetrics and Gynecology*.

[21] Conde-Agudelo A., Belizán J. M. (2000). Risk factors for pre-eclampsia in a large cohort of Latin American and Caribbean women. *British Journal of Obstetrics and Gynaecology*.

[22] Tranquilli A. L. (2014). Prediction, medical illness and the risk of pre-eclampsia. *Pregnancy Hypertension*.

[23] Kaze F. F., Njukeng F. A., Kengne A.-P. (2014). Post-partum trend in blood pressure levels, renal function and proteinuria in women with severe preeclampsia and eclampsia in Sub-Saharan Africa: a 6-months cohort study. *BMC Pregnancy and Childbirth*.

[24] Amaral L. M., Cunningham M. W., Cornelius D. C., LaMarca B. (2015). Preeclampsia: long-term consequences for vascular health. *Vascular Health and Risk Management*.

[25] Grubb A. O. (2000). Cystatin C-Properties and use as diagnostic marker. *Advances in Clinical Chemistry*.

[26] Padma Y., Aparna V. B., Kalpana B. (2013). Renal markers in normal and hypertensive disorders of pregnancy in Indian women: a pilot study. *International Journal of Reproduction, Contraception, Obstetrics and Gynecology*.

[27] Isasari K. M., Pangemanan W. T., Zulqarnain I., Rahadiyanto K. (2014). Maternal Cystatin C serum is higher in women with severe Preeclampsia. *Indones J Obs Gynecol*.

[28] Back S., Krutzen E., Nilsson-Ehle P. (2009). Contrast media as markers for glomerular filtration: a pharmacokinetic comparison of four agents. *Scandinavian Journal of Clinical and Laboratory Investigation*.

[29] Cataldi L., Mussap M., Bertelli L., Ruzzante N., Fanos V., Plebani M. (1999). Cystatin C in healthy women at term pregnancy and in their infant newborns: Relationship between maternal and neonatal serum levels and reference values. *American Journal of Perinatology*.

[30] Knight E. L., Verhave J. C., Spiegelman D. (2004). Factors influencing serum cystatin C levels other than renal function and the impact on renal function measurement. *Kidney International*.

[31] Kumaresan R., Giri P. (2011). A comparison of serum cystatin C and creatinine with glomerular filtration rate in indian patients with chronic kidney disease. *Oman Medical Journal*.

[32] Mikic A. N., Cabarkapa V., Nikolic A. (2012). Cystatin C in pre-eclampsia. *Journal of Maternal-Fetal and Neonatal Medicine*.

[33] Redman C. W., Beilin L. J., Bonnar J. (1976). Renal function in preeclampsia. *Journal of Clinical Pathology*.

[34] Redman C. W. G., Beilin L. J., Bonnar J., Wilkinson R. H. (1976). Plasma-urate measurements in predicting fetal death in hypertensive pregnancy. *The Lancet*.

[35] Schuster E., Weppelmann B. (1981). Plasma urate measurements and fetal outcome in preeclampsia. *Gynecologic and Obstetric Investigation*.

[36] Sagen N., Haram K., Nilsen S. T. (1984). Serum Urate as a Predictor of Fetal Outcome in Severe Pre‐Eclampsia. *Acta Obstetricia et Gynecologica Scandinavica*.

[37] Conrad K. P., Lindheimer M. D. (1999). Renal and cardiovascular alterations. *Hypertensive disorders in pregnancy Stamford (CT)*.

[38] Shemesh O., Golbetz H., Kriss J. P., Myers B. D. (1985). Limitations of creatinine as a filtration marker in glomerulopathic patients. *Kidney International*.

[39] Levey A. S., Perrone R. D., Madias N. E. (1988). Serum creatinine and renal function. *Annual Review of Medicine*.

[40] Perrone R. D., Madias N. E., Levey A. S. (1992). Serum creatinine as an index of renal function: new insights into old concepts. *Clinical Chemistry*.

[41] Strevens H., Wide-Swensson D., Ingemarsson I. (2001). Blood pressure during pregnancy in a Swedish population; impact of parity. *Acta Obstetricia et Gynecologica Scandinavica*.

[42] Haram K., Svendsen E., Abildgaard U. (2009). The HELLP syndrome: clinical issues and management. A Review. *BMC Pregnancy and Childbirth*.

[43] Strevens H., Wide-Swensson D., Torffvit O., Grubb A. (2002). Serum cystatin C for assessment of glomerular filtration rate in pregnant and non-pregnant women. Indications of altered filtration process in pregnancy. *Scandinavian Journal of Clinical and Laboratory Investigation*.

